# Erythropoiesis and Red Cell Indices Undergo Adjustments during Pregnancy in Response to Maternal Body Size but not Inflammation

**DOI:** 10.3390/nu12040975

**Published:** 2020-04-01

**Authors:** Rodrigo Vega-Sánchez, Mari Cruz Tolentino-Dolores, Blanca Cerezo-Rodríguez, Georgette Chehaibar-Besil, María Eugenia Flores-Quijano

**Affiliations:** 1 Departamento de Nutrición y Bioprogramación, Instituto Nacional de Perinatología Isidro Espinosa de los Reyes, Ciudad de México CP. 11000, Mexico; vegarodrig@gmail.com (R.V.-S.); cruz_tolentino@yahoo.com.mx (M.C.T.-D.); 2 Departamento de Ciencias de la Salud, Universidad del Valle de México, Coyoacán, Ciudad de México CP. 04910, Mexico; blancris@hotmail.com; 3 Departamento de Salud, Universidad Iberoamericana, Lomas de Santa Fe, Ciudad de México CP. 01219, Mexico; georgette.chb@gmail.com

**Keywords:** erythropoietin, red cell indices, pregnancy, maternal obesity, inflammation, hepcidin, hemoglobin

## Abstract

During human pregnancy, iron requirements gradually increase, leading to higher amounts of erythropoietin (EPO) and reticulocytes, and changes in erythrocyte size and density. Women with pregestational obesity experience “obesity hypoferremia” during pregnancy, which alters iron homeostasis. In this study we aimed to describe the relationship between EPO and iron nutrition status during nonanemic pregnancy, and to explore whether obesity and inflammation influence erythropoiesis and red cell indices. We conducted a secondary analysis of a cohort followed throughout pregnancy. Participants were nonanemic women assigned to two study groups based on pregestational body mass index (pgBMI): adequate weight (AW, *n* = 53) or obesity (Ob, *n* = 40). All received a multivitamin supplement. At gestational ages (GA) 13, 21, 28 and 34, we measured hemoglobin and red cell indices with an ACT-5DIFF hematology counter, and reticulocyte percentage by manual cell counting. EPO, interleukin (IL–6) and markers of iron status, i.e., hepcidin, serum transferrin receptor (sTfr) and ferritin, were measured by ELISA. Bivariate correlations showed that EPO was positively associated with pgBMI, GA, sTfr and IL-6, but negatively associated with hepcidin, ferritin and hemoglobin, and unrelated to iron intake. Generalized linear models adjusted for confounding factors showed that EPO and erythrocyte concentrations were significantly higher in women in the Ob group, while mean corpuscular volume (MCV), mean corpuscular hemoglobin (MCH) and red cell distribution width (RDW) were lower; reticulocytes and mean corpuscular hemoglobin concentration (MCHC) were not different. Differences were not altered when controlling for inflammation (IL–6). These changes suggest that, in addition to altering iron metabolism, a larger maternal body size during pregnancy results in higher erythropoiesis without increasing hemoglobin, which is exhibited in the latter being distributed among more and smaller erythrocytes.

## 1. Introduction

Iron changes as pregnancy progresses. During the first-trimester, iron requirements decrease, since menstruation has ceased and early hemodynamic changes do not yet demand greater amounts of this element. From the second trimester on, however, iron requirements steadily increase as maternal red blood cell mass expands, and placental and fetal growth accelerates [1,2].

To meet the increasing physiological iron needs and achieve a higher erythropoiesis rate, hormones such as hepcidin and erythropoietin play an important homeostatic role in maintaining hemoglobin concentrations. Hepcidin has been widely described for its pivotal role in regulating systemic iron availability through a negative feedback mechanism involving dietary iron absorption, as well as iron mobilization from reserves [3,4]. Erythropoietin, in turn, stimulates erythropoiesis, which is the proliferation, survival and differentiation of erythroid precursors within adult bone marrow [5].

As pregnancy evolves and more iron is required, hepcidin production in the liver is downregulated, partly in response to diminishing iron stores and an increasing need for iron in the erythroid marrow. Simultaneously, erythropoietin (EPO) synthesis is upregulated as a result of the increased glomerular filtration rate of hemodiluted blood, which derives from increased renal oxygen consumption and EPO synthesis in the placenta [5].

As a result of the higher EPO levels and erythropoiesis rate, reticulocyte percentage increases and erythrocytes become larger and less dense, which is indicative of a younger red cell population [6,7]. This is reflected in some red cell indices, including mean corpuscular volume (MCV, a measure of the size of individual erythrocytes), red cell distribution width (RDW, which indicates the heterogeneity of such size), mean corpuscular hemoglobin (MCH, an indicator of the amount of hemoglobin per erythrocyte) and mean corpuscular hemoglobin concentration (MCHC, a measure of the amount of hemoglobin in a deciliter of erythrocytes) [8]. Changes in these indices include an increase in MCV and RDW, while MCHC is not modified [6,9].

Apart from the physiological changes that occur in the maternal hematopoietic system during gestation, other factors can also modulate hormonal homeostatic iron regulation, as well as erythrocyte proliferation and maturation. These include iron nutrition status at the beginning of pregnancy (amount of iron stored) and later on, iron intake from the diet and supplementation [1,2,10], folic acid and vitamin B12 status [11], the presence of inflammatory processes [12] and obesity [13].

We have previously described that women who have pregestational obesity experience “obesity hypoferremia” during pregnancy [14]; an iron deficiency phenotype which simultaneously features the hallmarks of iron deficiency, including decreased serum iron and higher soluble transferrin receptor (sTfr) concentrations, as well as signs of anemia of chronic disease (ACD) such as unaltered ferritin and hemoglobin concentrations [3]. 

In the present study, we complement our previous work by evaluating how obesity modifies erythropoietin and red cell indices. Our objectives are 1) to describe the relationship between erythropoietin and iron nutrition status; 2) to describe the influence of obesity and inflammation on erythropoietin concentration; and 3) to describe how pregnancy, obesity and erythropoietin concentrations influence red cell indices, taking into consideration folic acid and vitamin B12 nutrition status. 

## 2. Materials and Methods

### 2.1. Participants

This is a secondary analysis of data obtained from a cohort of women followed throughout pregnancy at the National Institute of Perinatology in Mexico City. The study protocol received approval by the Institute’s Research and Ethics Committee (authorization number: 212250–49531) and study participants gave written informed consent. 

Details about the study’s design, inclusion and exclusion criteria, as well as attrition rates, can be found in a previously published article [14]. We assigned participants to one of two study groups based on their pregestational body mass index (pgBMI) [15]: adequate weight (pgBMI = 18.5–24.9 kg/m^2^) or obesity (pgBMI ≥ 30 kg/m^2^). The pgBMI was calculated using self-reported pregestational weight and height measured when women were invited to participate (with a SECA 242 stadiometer).

### 2.2. Data Collection

Women attended four study visits at gestational weeks 13, 21, 28 and 34. On the first study visit, sociodemographic and reproductive health information was collected. From that visit onward, all women received the multivitamin Nutrivida, which supplies 30 mg of elemental iron, 400 µg folic acid and 2.6 µg cyanocobalamin (vitamin B12) daily. Total daily iron intake (mg/day) was estimated by adding dietary and supplemental iron as previously described [14]. Other information obtained was the presence of a common stomach, respiratory urinary or vaginal infection in between study visits. Information about abortion or the development of pregnancy complications (particularly gestational diabetes and preeclampsia) were obtained from medical records. 

### 2.3. Blood Sampling and Metabolite Analysis

Maternal peripheral blood samples were obtained at each study visit after an overnight fast. Hematologic values were quantified from whole blood shortly after sample collection using an automated hematology counter (ACT–5DIFF, Beckman Coulter, Miami, FL, USA). These included hemoglobin (Hb), mean corpuscular volume (MCV), red cell distribution width (RDW) mean corpuscular hemoglobin (MCH) and mean corpuscular hemoglobin concentration (MCHC). 

Reticulocyte count was obtained by manual cell counting in a Neubauer chamber using supravital staining with 1% methylene blue. Reticulocytes are expressed as a percentage of total red blood cells. This count was additionally corrected with a normal hematocrit value (45%), thus obtaining the corrected reticulocyte percentage [16].

Blood serum was obtained by centrifuging whole blood for 10 min at 3500 rpm, which was aliquoted into microtubes and stored at −70 °C until analysis. Inflammatory and iron biomarkers were quantified in blood serum using commercially available kits, according to the manufacturer’s instructions. Erythropoietin (EPO), interleukin 6 (IL–6), ferritin, vitamin B12 and folic acid (serum and erythrocyte folate) were measured by ELISA and revealed with chemiluminescence (Immulite1000, Siemens, New York, NY, USA). Soluble transferrin receptor (sTfr) (R&D Systems, Minneapolis, MN, USA) and bioactive hepcidin–25 (DRG–Diagnostics kit, Marburg, Germany) were quantified with colorimetric ELISAs. All assays showed coefficients of variation <10% (Supplementary Table S1). 

All sample processing, storage and analysis was conducted at the Nutrition Laboratory of the National Institute of Perinatology. Sample sources were blinded to laboratory technicians.

### 2.4. Statistical Analyse

Variable distributions were analyzed for normality using the Kolmogorov–Smirnov test. Comparisons between study groups were done with Student’s t-distribution, Mann–Whitney U or Fisher tests as appropriate. Data are expressed as mean ± standard deviation, frequency (%) or median (Mn) and interquartile ranges (IQR). 

To address the study objectives, the following statistical methods were used:

(1) To identify the relationship between EPO and independent factors such as iron intake, pgBMI, gestational week and the presence of inflammation, as well as the relationship of EPO with hepcidin and iron nutrition status indicators. Pearson bivariate correlations were carried out using log-transformed data for variables without normal distribution.
(2) Differences in EPO along pregnancy between the AW and Ob groups were analyzed using two generalized linear models (GLM):

**Model 1:**

This model aimed to evaluate differences in EPO concentrations between study groups. Study group (Ob and AW), gestational age, presence of an underlying health condition (yes/no), presence of abortion or pregnancy complications (yes/no) and total iron intake were included as fixed factors in this model. Ferritin was included as a covariate. 

**Model 2:**

This model aimed to control for inflammation and see if it modified the difference in EPO concentrations between the groups. For this, we included IL–6 as another covariate in addition to those considered in Model 1.

(3) Differences in red cell indices between study groups were also analyzed using GLM. These models included study group (Ob and AW), gestational age, presence of an underlying health condition (yes/no) and presence of pregnancy complications (yes/no) as fixed factors, and vitamin B12 and EPO as covariates. As with EPO, models for red cell indices were performed with and without adding IL–6 to control for inflammation.

All statistical analyses were performed using SPSS v. 21 (IBM Inc., Chicago, IL, USA).

## 3. Results

A total of 93 women constituted the study sample, of which 40 were assigned to the obesity group (Ob) and 53 to the adequate weight (AW) group. Most sociodemographic characteristics were comparable between groups, although women in the Ob group tended to have higher parity and lower socioeconomic status. The sample characteristics, attrition rates throughout the study and iron and inflammatory concentrations on the first study visit have been published previously [14]. Serum and erythrocyte folate, vitamin B12 and erythropoietin concentrations, as well as erythrocyte count and characteristics, are shown in Table 1. 

### 3.1. Relationship between Erythropoietin and Other Factors

**Figure 1:**
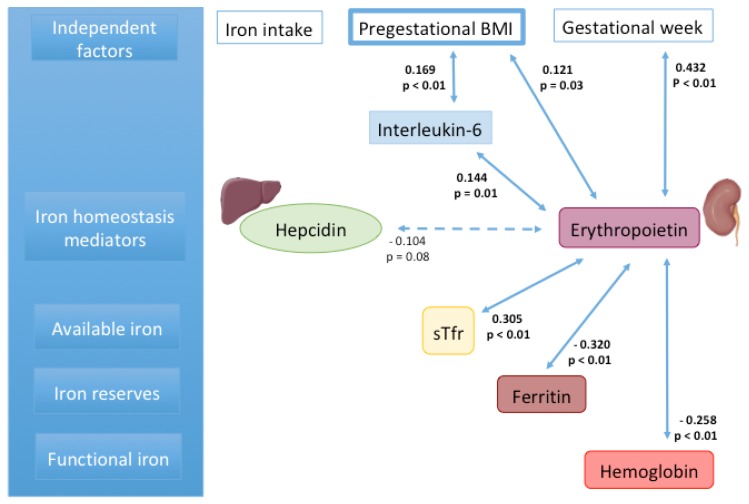
Bivariate associations. Pearson correlation among all variables (logarithmic transformation was used for variables with free distribution), r values and statistical significance are shown; continuous lines = *p* values <0.05; dotted lines = *p* values between >0.05 and <0.1.

Erythropoietin was positively associated with pg-BMI and gestational age and unrelated to iron intake. IL–6 was positively associated with pgBMI and EPO. Hepcidin (marginally), ferritin and hemoglobin were inversely associated with EPO, while sTfr was directly correlated with it. As expected, EPO was unrelated to folate and vitamin B12 concentrations (Figure 1).

### 3.2. Erythropoietin Concentration Differences between Obesity and Normal Weight Study Groups

**Figure 2:**
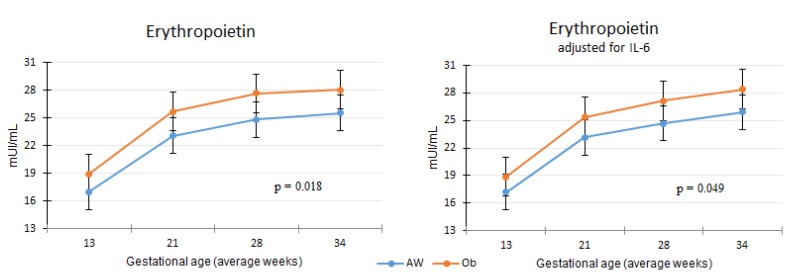
Erythropoietin concentration between adequate weight (AW) and obesity (Ob) groups.

The erythropoietin concentration was higher in the Ob group controlling for confounding variables such as ferritin, gestational age, underlying health conditions and pregnancy complications (gestational diabetes or preeclampsia). The difference remained significant when IL–6 was also included in the model (Figure 2).

### 3.3. Differences in Red Cell Indices between Obesity and Normal Weight Study Groups

Regarding red cells and red cell indices, reticulocyte count rose significantly from week 21 onward. The erythrocyte count showed a U shape behavior, as its concentration declined by week 21, remained low at week 27 and returned to its initial concentration by the end of the study follow-up. MCV increased as pregnancy progressed, while MCHC declined; MCH and RDW were not modified throughout pregnancy. Cross-sectional differences at study visits between groups were significant for erythrocytes, MCV, MCH and RDW. The values for all indices at each study visit can be found in Supplementary Table S2.

**Figure 3:**
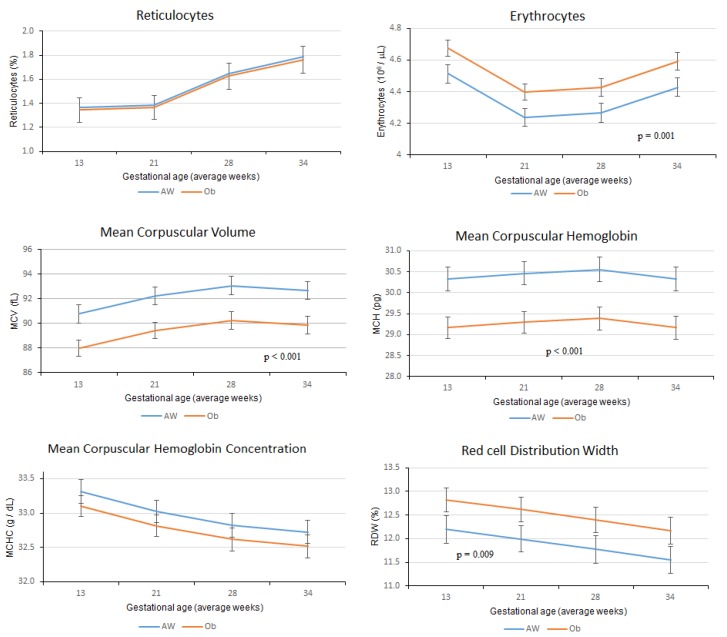
Differences in red cell indices between adequate weight (AW) and obesity (Ob) study groups. Adjusting models for inflammation by adding IL–6 did not alter the observed differences.

Comparisons of red cell indices between study groups took into account confounding variables such as health conditions at recruitment, gestational age, pregnancy complications, erythropoietin and vitamin V12. Erythrocyte concentrations were significantly higher in women in the Ob group, while MCV, MCH and RDW were lower, and MCHC was not different. Interestingly, the reticulocyte percentage showed no difference between groups (Figure 3).

Adding IL–6 to these models in order to adjust for inflammation did not alter the observed differences between groups. All models can be found in Supplementary Table S3.

## 4. Discussion

During human pregnancy, maternal erythropoietin is associated with iron nutrition status. Our results show that lower iron availability (as indicated by lower ferritin, higher sTfr, and lower hemoglobin) is associated with higher EPO concentration. However, no association was found between EPO and hepcidin, as documented in other studies [18]. We also found no association with iron intake, which is explained by the fact that all the women in our study were equally supplemented. As expected, EPO was related to gestational age, since with the progress of pregnancy, blood volume increases and iron reserves normally decrease.

The central finding of our study was that EPO concentration is higher in women with obesity, even when controlling for iron status and inflammation. Few other studies have explored the influence of obesity on EPO concentration; one study in adolescents [19] and another in Mexican adult women, which controlled for inflammation [20]. These studies found no differences in EPO between individuals with adequate weight or obesity. 

Higher EPO concentration among pregnant women with larger bodies may be a response to two concomitant mechanisms: First, the anti-inflammatory and cytoprotective role of EPO against hypoxia-induced inflammation of the adipose tissue resulting from obesity [21,22]; and second, having a larger body results in increased need of iron to meet greater tissue oxygen demands.

However, although EPO concentrations were higher in women in the Ob group, its profiles as the pregnancies progressed were not different than those of the women in the AW group. This supports the observation that obesity, inflammation and iron reserves do not affect EPO regulation during pregnancy. Moreover, this is also reflected in erythrocyte concentrations, which show the same profile as EPO.

The fact that higher concentrations of EPO were observed in the obesity group may suggest a physiological compensatory mechanism for ensuring adequate erythropoiesis in cases with low available iron, such as pregnant women with obesity or those in need of iron supplementation. Whether such a mechanism would result in pregnant women benefiting from obesity, in terms of erythropoiesis maintenance, is an hypothesis worth exploring. Such obesity-associated advantages have recently been reported in people who undergo hemodialysis [23].

In addition to reporting EPO values, our study adds knowledge about red cell indices during pregnancy, which is somewhat scarce. While other authors have published information about how these indices change [6,9], we provide evidence concerning their differences between women who start pregnancy with adequate weight or obesity, and whether inflammation alters such differences.

We have previously reported that hemoglobin concentrations do not change according to body size [14]. Correspondingly, MCHC is not different between AW and Ob groups. However, MCV and MCH are lower in the Ob group; this is simply a dilution effect, since the same amount of hemoglobin is distributed among more erythrocytes. Moreover, this distribution is not homogeneous among erythrocytes, as indicated by higher RDW.

One of our initial hypotheses was that a larger number of immature erythrocytes would be found in women in the Ob group, since increasing EPO results in lower red cell maturation time, increased speed of hemoglobin synthesis and early release of reticulocytes from bone marrow [8]. However, this hypothesis was proven to be wrong, as shown by the lack of difference in reticulocytes between the study groups. This implies that pregnancy involves physiological adjustments to red cell production in order to meet the required amount of mature erythrocytes, regardless of maternal body size and inflammation.

Finally, it should be noted that we considered ferritin and sTFr in our assessments of iron status based on the WHO guidelines [24]. Several international guidelines also recommend ferritin for iron deficiency (ID) diagnoses [25]. However, since ferritin is an acute-phase protein, it may not be the most reliable indicator of iron need, especially in the presence of inflammatory conditions. In this sense, transferrin saturation (TSAT) has been proposed as an alternative, using a <20 % value as cut off. However, TSAT is not as efficient as ferritin in diagnosing early stage iron deficiency, and it alone cannot differentiate absolute from functional ID [26]. Therefore, using both ferritin and TSAT combined would result in a better diagnostic capability for ID. Further studies are required to define the physiological ranges of TSAT during pregnancy and to evaluate the <20 % threshold for diagnosing pregnancy-related anemia.

Taking into account our previous results and the current ones, we conclude that even when pregnancy starts with an adequate iron nutrition status and supplementation, a larger maternal body size entails changes in available iron resulting in hypoferremia and increased erythropoiesis. Once the amount of available iron for hemoglobin production is established, the latter is adjusted to the greater number of circulating erythrocytes. Altogether, our results suggest that, while larger maternal body size and inflammation alter iron homeostasis during pregnancy, the mechanisms involved in red cell function seem to be carefully maintained so as not to be affected by such factors. 

## Figures and Tables

**Table 1:** Erythropoietin, red cell indices, iron status and inflammatory biomarkers at the first study visit.

	Adequate Weight (*n* = 53)	Obese (*n* = 40)	*p*
Erythropoietin	16.00 (12.95, 19.10)	18.05 (13.65, 23.07)	0.19
Reticulocytes (% of total erythrocytes, corrected with hematocrit)	1.20 (1.00, 1.87)	1.40 (1.00, 1.80)	0.77
Erythrocytes (10^6^/µL)	4.50 (4.34, 4.73)	4.54 (4.40, 4.98)	0.20
MCV (fL)	90.86 (88.30, 94.23)	88.88 (85.42, 92.54)	0.05
MCH (pg)	30.30 (29.28, 31.42)	29.31 (27.93, 30.80)	<0.01
MCHC (g/dL)	33.32 (32.76, 34.03)	33.01 (32.43, 33.80)	0.14
RDW	11.69 (11.06, 12.60)	12.23 (11.51, 13.28)	0.02
Hemoglobin (g/dL)	13.55 (13.18, 14.45)	13.39 (13.08, 13.99)	0.36
Ferritin (ng/mL)	39.30 (27.60, 65.05)	40.60 (19.40, 96.15)	0.89
sTfr (mg/L)	1.00 (0.84, 1.20)	1.04 (0.82, 1.44)	0.41
Serum iron (μg/dL)	162.45 (129.8, 199.5)	149.76 (113.6, 199.7)	0.47
Hepcidin (ng/mL)	8.04 (5.88, 11.86)	9.58 (6.21, 15.67)	0.23
Serum folate (ng/mL)	31.90 (24.30, 38.30)	27.90 (24.32, 41.75))	0.28
<3 ng/mL *	0	0	
Erythrocyte folate (pg/mL)	567.0 (464.5, 748.65)	523.0 (368.5, 778.0)	0.58
<120 pg/mL *	0	0	
Vitamin B12 (pg/mL)	344.50 (263.75, 494.50)	258.50 (186.75, 352.25)	<0.01
<200 pg/mL *	5 (10.5%)	10 (26.3%)	0.05
IL–6 (pg/mL)	1.79 (1.63, 2.10)	2.15 (1.81, 2.43)	<0.01
Leptin (pg/mL)	21.50 (15.11, 26.25)	44.48 (32.14, 61.57)	<0.01

Values are median (IQR), compared using Mann–Whitney U test. Significant differences (*p* < 0.05) are noted in bold font. * Deficiency cutoff values [17].

## Supplementary Materials

The following are available online at https://www.mdpi.com/2072-6643/12/4/975/s1, Table S1. Intra-assay coefficients of variation, Table S2. Red cell indices at each study visit, Table S3. Generalized Linear Models for erythropoietin and red cell indices.

## References

[1] Bothwell T.H. (2000). Iron requirements in pregnancy and strategies to meet them. Am. J. Clin. Nutr..

[2] Fisher A.L., Nemeth E. (2017). Iron homeostasis during pregnancy. Am. J. Clin. Nutr..

[3] Tussing-Humphreys L., Pusatcioglu C., Nemeth E., Braunschweig C. (2012). Rethinking iron regulation and assessment in iron deficiency, anemia of chronic disease, and obesity: Introducing hepcidin. J. Acad. Nutr. Diet..

[4] Ganz T., Nemeth E. (2012). Hepcidin and iron homeostasis. Biochim. Biophys. Acta.

[5] Conrad K.P., Benyo D.F., Westerhausen-Larsen A., Miles T.M. (1996). Expression of erythropoietin by the human placenta. FASEB J..

[6] Lurie S. (1993). Changes in age distribution of erythrocytes during pregnancy: A longitudinal study. Gynecol. Obstet. Investig.

[7] Lurie S., Mamet Y. (2000). Red blood cell survival and kinetics during pregnancy. Eur. J. Obstet. Gynecol. Reprod. Biol..

[8] McKenzie S.B. (2000). Hematología Clínica.

[9] Bolton F.G., Street M.J., Pace A.J. (1983). Changes in Erythrocyte Volume and Shape in Pregnancy. Obstet. Gynecol. Surv..

[10] Koenig M., Tussing-Humphreys L., Day J., Cadwell B., Nemeth E. (2014). Hepcidin and Iron Homeostasis during Pregnancy. Nutrients.

[11] Hall J.E. (2015). Guyton and Hall Textbook of Medical Physiology E-Book.

[12] Sangkhae V., Nemeth E. (2017). Regulation of the Iron Homeostatic Hormone Hepcidin. Adv. Nutr..

[13] Yanoff L.B., Menzie C.M., Denkinger B., Sebring N.G., McHugh T., Remaley A.T., Yanovski J.A. (2007). Inflammation and iron deficiency in the hypoferremia of obesity. Int. J. Obes..

[14] Flores-Quijano M.E., Vega-Sánchez R., Tolentino-Dolores M.C., López-Alarcón M.G., Flores-Urrutia M.C., López-Olvera A.D., Talavera J.O. (2019). Obesity Is Associated with Changes in Iron Nutrition Status and Its Homeostatic Regulation in Pregnancy. Nutrients.

[15] Obesity and Overweight. http://www.who.int/mediacentre/factsheets/fs311/en/.

[16] Crosby W.H. (1981). Reticulocyte counts. Arch. Intern. Med..

[17] Gibson R.S. (2005). Principles of Nutritional Assessment.

[18] Schulze K.J., Christian P., Ruczinski I., Ray A.L., Nath A., Wu L.S.-F., Semba R.D. (2008). Hepcidin and iron status among pregnant women in Bangladesh. Asia Pac. J. Clin. Nutr..

[19] Cao C., Pressman E.K., Cooper E.M., Guillet R., Westerman M., O’Brien K.O. (2016). Prepregnancy Body Mass Index and Gestational Weight Gain Have No Negative Impact on Maternal or Neonatal Iron Status. Reprod. Sci..

[20] Flores-Quijano M.E., Montalvo-Velarde I., Vital-Reyes V.S., Rodríguez-Cruz M., Rendón-Macías M.E., López-Alarcón M. (2016). Longitudinal Analysis of the Interaction between Obesity and Pregnancy on Iron Homeostasis: Role of Hepcidin. Arch. Med. Res..

[21] Villarroel P., Arredondo M., Olivares M. (2013). Anemia de las enfermedades crónicas asociada a obesidad: Papel de la hepcidina como mediador central. Rev. Méd. Chile.

[22] Kowalska-Kańka A., Maciejewski T., Niemiec K.T. (2013). The role and regulation of secretion of erythropoietin in pregnancy. Med. Wieku Rozwoj..

[23] El-Kannishy G.M., Megahed A.F., Tawfik M.M., El-Said G., Zakaria R.T., Mohamed N.A., Taha E.M., Ammar A.A., Abd Eltawab A.M., Sayed-Ahmed N.A. (2018). Obesity may be erythropoietin dose-saving in hemodialysis patients. Kidney Res. Clin. Pract..

[24] WHO (2014). Serum Transferrin Receptor Levels for the Assessment of Iron Status and Iron Deficiency in Populations.

[25] Peyrin-Biroulet L., Williet N., Cacoub P. (2015). Guidelines on the diagnosis and treatment of iron deficiency across indications: A systematic review. Am. J. Clin. Nutr..

[26] Elsayed M.E., Sharif M.U., Stack A.G., Makowski G.S. (2016). Transferrin Saturation: A Body Iron Biomarker. Advances in Clinical Chemistry.

