# Supplementary material for: Erythropoiesis and Red Cell Indices Undergo Adjustments during Pregnancy in Response to Maternal Body Size but not Inflammation
Source: Nutrients. 2020 Apr 1;12(4):975. doi: 10.3390/nu12040975 (PMC7230988; doi:10.3390/nu12040975)
Supplement: Supplementary file 1 [file nutrients-12-00975-s001.zip › Table S3. Generalized Linear Models for erythropoietin and red cell indices.pdf]

Table S3. Generalized Linear Models for erythropoietin and red cell indices

[a] compared to group AW, [b] compared to 34 weeks, [c] absence of underlying health conditions or pregnancy complications.

ERYTHROPOIETIN PREDICTIVE PARAMETERS

		Model 1			Model 2	
	β	95% CI	p	β	95% CI	p
Intercept	3.646	3.385, 3.908	<0.01	3.610	3.332, 3.888	<0.01
Ob[a]	0.108	0.197, 0.018	0.01	0.093	0.186, 0.001	0.04
Gestational age (average) 13w[b]	-0.407	-0.539, -0.276	<0.01	-0.410	-0.545, -0.275	<0.01
21w[b]	-0.102	-0.230, 0.026	0.117	-0.112	-0.243, 0.019	0.09
28w[b]	-0.029	-0.159, 0.101	0.66	-0.047	-0.180, 0.086	0.48
Underlying[c]	0.020	-0.067, 0.107	0.65	0.023	-0.065, 0.112	0.60
Complication[c]	-0.065	-0.196, 0.067	0.33	-0.065	-0.201, 0.072	0.35
Ferritin-Ln	-0.084	-016, - 0.021	<0.01	-0.092	-0.156, - 0.027	<0.01
IL-6-Ln				0.074	-0.019, 0.167	0.19
R^2 (R^2 adjusted)	0.265 (0.247)			0.275 (0.253)		

RED CELL INDICES PREDICTIVE PARAMETERS

Reticulocytes							
	β	95% CI	p		β	95% CI	p
Intercept	2.092	1.241, 2.943	<0.01		1.861	0.976, 2.745	<0.01
Ob[a]	-0.021	-0.163, 0.206	0.82		0.033	-0.155, 0.221	0.73
Gestational age (average) 13w[b]	-0.420	-0.675,-0.165	<0.01		-0.396	-0.656, -0.137	<0.01
21w[b]	-0.399	-0.634, -0.164	<0.01		-0.357	-0.599, --0.115	<0.01
28w[b]	-0.138	-0.382,0.105	0.26		-0.128	-0.375, 0.119	0.31
Underlying[c]	-0.041	-0.20, 0.124	0.62		-0.074	-0.242, 0.093	0.38
Complication[c]	-0.161	-0.427, 0.106	0.23		-0.093	-0.372, 0.186	0.51
Erythropoietin-Ln	-0.062	-0.298 ,0.173	0.60		-0.056	-0.293, 0.181	0.64
Vitamin B12	0.000		0.57		0.000		0.43
IL-6-Ln					0.214	0.018, 0.411	0.03
R^2 (R^2 adjusted)	0.084 (0.048)				0.101 (0.059)		

Erithrocytes							
	β	95% CI	p		β	95% CI	p
Intercept	5.358	4.939, 5.778	<0.01		5.195	4.764, 5.625	<0.01
Ob[a]	0.162	0.068, 2.56	<0.01		-0.146	-0.241, -0.051	<0.01
Gestational age (average) 13w[b]	0.085	-0.046, 0.216	0.20		0.107	-0.026, 0.239	0.11
21w[b]	-0.192	-0.313, -0.072	<0.01		-0.204	-0.326, -0.082	<0.01
28w[b]	-0.162	-0.286, -0.039	0.01		-0.154	-0.279, -0.030	0.01

Underlying[c]	0.039	-0.045, 0.124	0.36		0.037	-0.049, 0.122	0.396
Complication[c]	-0.184	-0.318, -0.050	<0.01		-0.138	-0.276, 0.000	0.05
Erythropoietin-Ln	-0.224	-0.339, -0.109	<0.01		-0.211	-0.326, -0.097	<0.01
Vitamin B12	0.000		0.93		0.000		0.91
IL-6					0.094	-0.004, 0.192	0.06
R^2 (R^2 adjusted)	0.286 (0.261)				0.304 (0.275)		

**Mean Corpuscular Volume (MCV)**							
	**β**	**95% CI**	**p**		**β**	**95% CI**	**p**
Intercept	82.870	77.563, 88.178	<0.01		84.416	78.854, 89.978	<0.01
Ob[a]	2.821	1.636, 4.007	<0.01		2.674	1.448, 3.900	<0.01
Gestational age (average) 13w[b]	-1.897	-3.552, -0.242	0.02		-2.126	-3.839, -0.414	0.01
21w[b]	-0.44	-1.963, 1.079	0.56		-0.414	-1.994, 1.165	0.60
28w[b]	0.394	-1.171, 1.960	0.62		0.310	-1.295, 1.915	0.70
Underlying[c]	-0.668	-1.739, 0.403	0.22		-0.608	-1.713, 0.496	0.28
Complication[c]	1.338	-0.360, 3.035	0.12		0.262	-0.765, 2.801	0.26
Erythropoietin-Ln	1.983	0.532, 3.434	<0.01		1.797	0.315, 3.280	0.02
Vitamin B12	0.001		0.21		0.166	-0.001, 0.004	0.17
IL-6					-0.768	-2.035, 0.498	0.23
R^2 (R^2 adjusted)	0.245 (0.218)				0.244 (0.212)		

**Mean Corpuscular Hemoglobin (MCH)**							
	**β**	**95% CI**	**p**		**β**	**95% CI**	**p**
Intercept	27.025	24.95, 29.095	<0.01		27.458	25.279, 29.637	<0.01
Ob[a]	-1.161	0.698, 1.623	<0.01		1.124	0.643, 1.604	<0.01
Gestational age (average) 13w[b]	0.001	-0.645, 0.647	0.99		-0.006	-0.677, 0.665	0.99
21w[b]	0.131	-0.462, 0.724	0.66		0.135	-0.484, 0.754	0.67
28w[b]	-0.224	-0.386, 0.835	0.47		0.195	-0.434, 0.824	0.54
Underlying[c]	-0.179	-0.597, 0.239	0.39		-0.143	-0.576, 0.289	0.51
Complication[c]	0.737	0.074, 1.399	0.02		0.646	-0.053, 1.344	0.07
Erythropoietin-Ln	0.561	-0.005, 1.227	0.05		0.554	-0.027, 1.135	0.062
Vitamin B12	0.000		0.45		0.000		0.39
IL-6					-0.423	-0.919, 0.073	0.09
R^2 (R^2 adjusted)	0.193 (0.164)				0.196 (0.162)		

**Mean Corpuscular Hemoglobin Conentration (MCHC)**							
	**β**	**95% CI**	**p**		**β**	**95% CI**	**p**
Intercept	32.715	31.471, 33.960	<0.01		32.625	31.325, 33.926	<0.01
Ob[a]	0.208	-0.070, 0.486	0.14		0.216	-0.070, 0.503	0.14
Gestational age (average) 13w[b]	0.587	0.199, 0.975	<0.01		0.652	0.252, 1.053	<0.01

21w[b]	0.299	-0.058, 0.655	0.10		0.296	-0.073, 0.665	0.12
28w[b]	0.099	-0.268, 0.466	0.59		0.095	-0.280, 0.470	0.62
Underlying[c]	-0.050	-0.301, 0.201	0.69		-0.040	-0.298, 0.218	0.76
Complication[c]	0.269	-0.129, 0.667	0.18		0.283	-0.133, 0.700	0.19
Erythropoietin-Ln	-0.79	-0.419, 0.262	0.65		-0.022	-0.369, 0.325	0.90
Vitamin B12	0.000		0.47		0.000		0.48
IL-6					0.298	-0.453, 0.139	0.30
R^2 (R^2 adjusted)	0.086 (0.053)				0.100 (0.062)		

Red cell Distribution Width (RDW)							
	**β**	**95% CI**	**p**		**β**	**95% CI**	**p**
Intercept	12.815	10.731, 14.898	<0.01		12.068	9.961, 14.175	<0.01
Ob[a]	-0.620	-1.086, -0.154	<0.01		-0.509	-0.974, -0.044	0.03
Gestational age (average) 13w[b]	0.651	-0.004, 1.305	0.05		0.786	0.132, 1.440	0.02
21w[b]	0.448	-145, 1.041	0.13		0.453	-0.141, 1.047	0.13
28w[b]	0.229	-0.379, 0.837	0.46		0.280	-0.321, 0.882	0.36
Underlying[c]	0.391	-0.029, 0.812	0.06		0.401	-0.017, 0.820	0.06
Complication[c]	-0.372	-1.034, 0.289	0.269		-0.184	-0.854, 0.487	0.59
Erythropoietin-Ln	-0.214	-0.784, 0.357	<0.01		-0.103	-0.666, 0.459	0.72
Vitamin B12	0.000		0.94		0.000		0.95
IL-6					0.142	-0.333, 0.618	0.55
R^2 (R^2 adjusted)	0.099 (0.066)				0.093 (0.054)		
