# Supplementary material for: Erythropoiesis and Red Cell Indices Undergo Adjustments during Pregnancy in Response to Maternal Body Size but not Inflammation
Source: Nutrients. 2020 Apr 1;12(4):975. doi: 10.3390/nu12040975 (PMC7230988; doi:10.3390/nu12040975)
Supplement: Supplementary file 1 [file nutrients-12-00975-s001.zip › Table S1. Intra-assay coefficients of variation..pdf]

Table S1. Intra-assay coefficients of variation.

Analyte	Coefficient of variation (%)
Erythropoietin<br>(ref: LKEP1, Siemens)	5.1 - 8.1
Interleukin 6<br>(ref: LK6P1, Siemens)	3.5 - 6.2
Ferritin<br>(ref: LKFE1, Siemens)	3.9 - 6.5
Serum transferrin receptor<br>(ref: DTFR1, R&D Systems)	4.3 - 7.1
Hepcidin<br>(ref: EIA-5258, DRG-Diagnostics)	2.1 - 9.9
Vitamin B12<br>(ref: LKVB1, Siemens)	5.9 - 11.3
Folic acid<br>(LKFO1, Siemens)	5.2 - 8.8
