# Supplementary material for: Erythropoiesis and Red Cell Indices Undergo Adjustments during Pregnancy in Response to Maternal Body Size but not Inflammation
Source: Nutrients. 2020 Apr 1;12(4):975. doi: 10.3390/nu12040975 (PMC7230988; doi:10.3390/nu12040975)
Supplement: Supplementary file 1 [file nutrients-12-00975-s001.zip › Table S2. Red cell indices at each study visit..pdf]

**Table S2. Red cell indices comparison between study groups at each study visit.**

| | Visit 1 | | | Visit 2 | | | Visit 3 | | | Visit 4 | | |
|---|---|---|---|---|---|---|---|---|---|---|---|---|
| | **AW (n=53)** | **Ob (n=40)** | **p** | **AW (n=42)** | **Ob (n=36)** | **p** | **AW (n=38)** | **Ob (n=32)** | **p** | **AW (n=33)** | **Ob (n=23)** | **p** |
| **Erythrocytes ($10^6$/μL)** | 4.5±0.37 | 4.64±0.38 | 0.26 | 4.15±0.34 | 4.35±0.38 | 0.02 | 4.17±0.29 | 4.31±0.31 | 0.07 | 4.30±0.31 | 4.51±0.29 | 0.01 |
| **Reticulocytes (%)** | 1.43±0.58 | 1.45±0.56 | 0.90 | 1.48±0.61 | 1.76±0.81 | 0.11 | 1.84±0.75 | 1.95±2.09 | 0.79 | 1.90±.056 | 1.81±0.61 | 0.59 |
| **MCV (fL)** | 90.40±4.91 | 88.69 ± 4.94 | 0.10 | 92.91±4.60 | 89.83±3.95 | <0.01 | 93.91±3.68 | 90.01±3.30 | <0.01 | 93.63±3.90 | 90.01±3.30 | <0.01 |
| **MCH (pg)** | 30.34±2.00 | 29.29±2.10 | 0.01 | 30.71±1.61 | 29.68±1.43 | <0.01 | 30.97±1.65 | 29.24±1.20 | <0.01 | 30.79±1.45 | 29.24±1.20 | <0.01 |
| **MCHC (g/dL)** | 33.43±1.21 | 33.02±1.24 | 0.10 | 33.05±0.60 | 33.04±0.92 | 0.92 | 32.96±0.89 | 32.71±0.82 | 0.22 | 32.88±0.81 | 32.49±0.73 | 0.70 |
| **RDW (%)** | 12.11±1.91 | 12.53±1.40 | 0.24 | 11.65±1.23 | 12.73±1.61 | <0.01 | 11.64±1.23 | 12.03±1.20 | 0.28 | 11.33±1.65 | 12.18±1.48 | 0.02 |

AW = adequate weight; Ob = obesity. MCV = mean corpuscular volume, MCH = mean corpuscular hemoglobin, MCHC = mean corpuscular hemoglobin concentration, RDW = red cell distribution width. Values represent mean concentrations ± s.d. Statistical differences using T-test.
